# Investigation of light delivery geometries for photoacoustic applications using Monte Carlo simulations with multiple wavelengths, tissue types, and species characteristics

**DOI:** 10.1117/1.JBO.25.1.016005

**Published:** 2020-01-23

**Authors:** Timothy Sowers, Heechul Yoon, Stanislav Emelianov

**Affiliations:** a Georgia Institute of Technology, Parker H. Petit Institute for Bioengineering and Bioscience, Atlanta, Georgia, United States; b Georgia Institute of Technology, George W. Woodruff School of Mechanical Engineering, Atlanta, Georgia, United States; c Georgia Institute of Technology, School of Electrical and Computer Engineering, Atlanta, Georgia, United States; d Emory University School of Medicine and Georgia Institute of Technology, Wallace H. Coulter Department of Biomedical Engineering, Atlanta, Georgia, United States

**Keywords:** photoacoustics, ultrasound, clinical, imaging, Monte Carlo, mouse

## Abstract

Combined ultrasound and photoacoustic imaging systems are being developed for biomedical and clinical applications. One common probe configuration is to use a linear transducer array with external light delivery to produce coregistered ultrasound and photoacoustic images. The diagnostic capability of these systems is dependent on the effectiveness of light delivery to the imaging target. We use Monte Carlo modeling to investigate the optimal design geometry of an integrated probe. Simulations are conducted with multiple tissue compositions and wavelengths. The effect of a skin layer with the thickness of a mouse or a human is also considered. The model was validated using a tissue-mimicking gelatin phantom and corresponding Monte Carlo simulations. The optimal illumination angle is shallower with human skin thickness, whereas intermediate angles are ideal with mouse skin thickness. The effect of skin thickness explains differences in the results of prior work. The simulations also indicate that even with identical hardware and imaging parameters, light delivery will be up to 3× smaller in humans than in mice, due to the increased scattering from thicker skin. Our findings have clear implications for the many researchers using mice to test and develop imaging methods for clinical translation.

## 1 Introduction

Photoacoustic (PA) imaging^1^^–^^6^ is an emergent area of research in biomedical imaging. The technique uses nanosecond pulses of light to induce ultrasonic waves from imaging targets in tissue, which can then be detected by an ultrasound (US) transducer. The ultrasonic wave is induced by rapid expansion of the target as a result of heat generated from the absorbed laser light. The strength of the ultrasonic wave is dependent on the quantity of light delivered, a set of physical properties referred together as the Gruneisen coefficient, and the optical absorption coefficient of the imaging target.^5^ Since the optical absorption coefficient is wavelength dependent and has been characterized for a wide variety of tissue types, imaging targets can be identified by imaging a single region at multiple wavelengths and then comparing the changes in PA signal intensity with what would be predicted based on the known optical absorption coefficients.

PA imaging is being widely developed for applications in the biomedical field, including, for example, the detection of various cancers and lymph node metastasis, detection of circulating tumor cells, identification of atherosclerotic plaques, evaluation of muscle oxygenation, identification of contrast agents, and temperature sensing.^6^^–^^13^ A significant benefit of PA imaging is that it can be used synergistically with other imaging techniques, including conventional B-mode US, flow imaging, and elasticity imaging.^14^ One common technique for PA image acquisition is to use a commercial linear US array with laser light delivered by an optical fiber.^15^^,^^16^ This method takes advantage of developments in commercial US transducer arrays to detect the ultrasonic PA waves, while making it possible to overlay the PA signal with anatomical information obtained from an US B-mode image. Examples of studies that specifically use commercial US arrays with external light delivery can be found in the literature,^8^^,^^9^^,^^17^^–^^19^ including one application currently in clinical trials for breast cancer diagnosis.^20^

Since the PA pressure generated at the imaging target is proportional to the laser fluence at that location, increased light delivery into the tissue will result in stronger PA signals from native absorbers. In addition, improved light delivery can enhance the use of PA contrast agents, which are being developed for contrast-enhanced imaging and super-resolution imaging.^21^^,^^22^ Various research groups have conducted studies to determine the optimal geometrical layout of the fiber bundle and transducer to increase light delivery. Haisch et al.^15^ proposed the use of a fiber bundle and commercial transducer. They determined experimentally that a 40-deg to 50-deg angle between the fiber bundle and transducer is best for imaging depths from 10 to 25 mm, while shallower angles are better at greater depths. The modeling of Wang et al.^23^ indicates that decreasing the distance between the fiber bundle and the transducer imaging plane will increase light delivery. However, in contrast to the results of Haisch et al.,^15^ they found that decreasing the angle between the fiber bundle and transducer will monotonically increase fluence along the imaging plane at depths of 5 to 30 mm. Sivasubramanian et al.^24^ modeled different combinations of the fiber bundle to transducer distance, fiber bundle to transducer angle, and the transducer to tissue distance to optimize the signal-to-noise ratio (SNR) and tested the results experimentally. It was found that different combinations of these parameters can result in large differences in SNR. Finally, Sangha et al.^25^ designed a motorized system in which the angle of the fiber bundle can be changed on demand during an experiment. This system was used to vary the light emission angle to separately optimize imaging of subcutaneous fat and periaortic fat in mice *in vivo*. They found differences between the optimal angle in their phantom, which had no skin-mimicking layer, and experiments conducted in mice.

In this paper, we extend these studies by investigating the differences in the optimal design that arise from the presence of skin with the thickness of a human or mouse. We hypothesize that the high scattering in this layer explains differences in the results of Haisch et al.,^15^ Wang et al.,^23^ and Sangha et al.^25^ We investigate the effect of changing the thickness of the skin layer at multiple wavelengths and with multiple soft tissue compositions below the skin using Monte Carlo simulations of photon propagation. This is of particular interest to researchers, since the use of mice for *in vivo* validation of preclinical techniques is common. We conduct experiments in a phantom at 1064 nm and compare the results with simulations from the Monte Carlo software for validation. We present different light emission and transducer configurations to maximize fluence delivery when using the skin thickness of a mouse versus the skin thickness of a human. We also find that differences in the optimal geometries between species can explain variability in the results of prior work. Importantly, the results indicate that significant variations in fluence can be expected between mice and humans using an identical imaging setup and imaging parameters, which serves as a cautionary message to researchers expecting to directly translate results in mice to the clinic.

## 2 Materials and Methods

### 2.1 Monte Carlo Modeling Software

MCXLAB, a package within Monte Carlo eXtreme^26^^–^^28^ that has been developed for use with MATLAB and GNU Octave, was used to simulate photon transport. The software simulates photon propagation from a defined source through a volume consisting of cubic voxels. The optical properties at each voxel are defined by the absorption coefficient, $\mu_{a}$, scattering coefficient, $\mu_{s}$, anisotropy, g, and index of refraction, n. In these simulations, all voxels were cubic with a length of 50  μm. The Monte Carlo software output makes it possible to attain the fluence in each voxel, normalized to the total energy from the light source. Thus, the output of the Monte Carlo simulation is the normalized fluence.

**Fig. 1:**
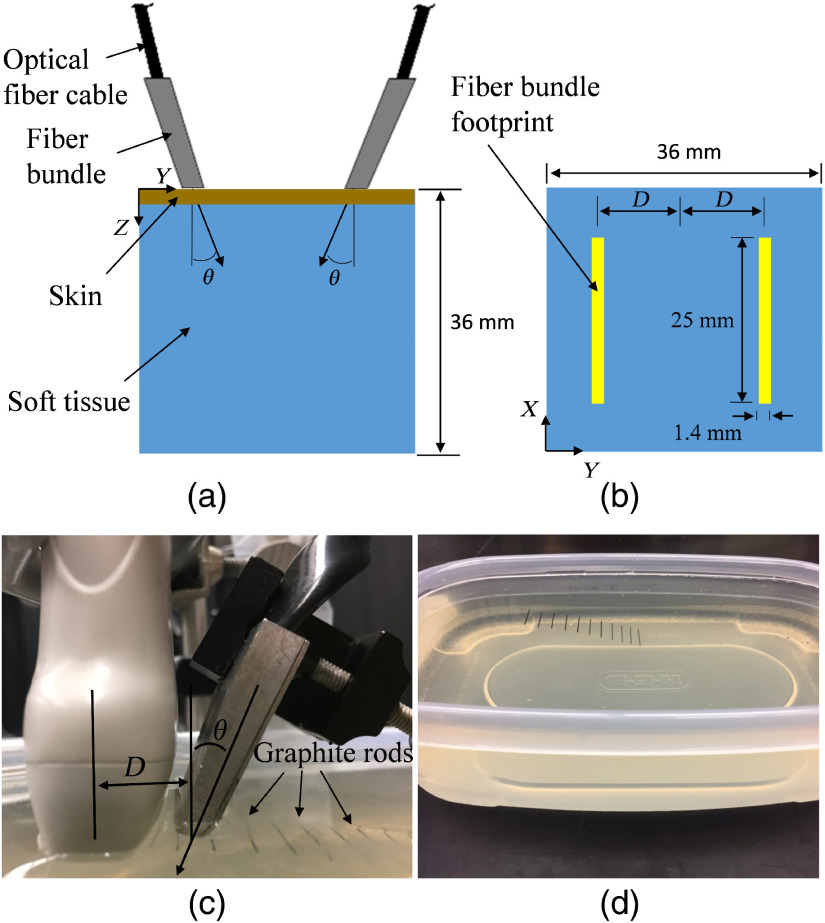
Diagram of the (a) (b) model and (c), (d) experimental setup. (a) A side view diagram of the tissue model geometry showing the location and orientation of the optical fibers used for light delivery. (b) A top view diagram of the tissue model geometry. The optical fibers have a rectangular footprint with dimensions of 25  mm×1.4  mm. In general, two cables of optical fibers are used for light delivery during PA imaging experimentally, although only one was needed for our model and experiment due to the geometrical symmetry. (c) The experimental setup with the location of light emission from the optical fibers separated from the transducer array by a distance, D, and at an angle, θ. (d) Graphite rods at various depths in the gelatin phantom were used as PA absorbers. The rods all had a length of 2 cm and a diameter of 0.5 mm.

Combined US/PA systems that use a commercial US transducer with optical fibers for delivery of laser irradiation generally illuminate the imaging target with the distal end of the optical fibers positioned on each side of the transducer. In these simulations, the light emission from the distal end of the optical fibers was simulated as a light source of uniform fluence with an area of $25\times 1.4\;{\mathrm{mm}}^{2}$. The center of the light emission area was offset from the center of the simulation space (in practice, the imaging plane of the transducer array) by a distance, D [Figs. 1(a) and 1(b)]. The light was emitted into the simulated volume at a uniform angle, θ. Since the optical fiber and transducer array setup is symmetrical across the imaging plane of the US transducer, only one light source needed to be simulated. After the simulation, the average of the fluence output and its mirror across the imaging plane was used as the final fluence output. The boundary condition at the edge of the simulation volume was set such that any photons that crossed the boundary permanently escaped the simulation volume.

### 2.2 Modeling Parameters

The mouse and human tissue geometries were simulated using multiple tissue types and wavelengths (Table 1). Simulations were conducted using both fibrous^34^^,^^35^ and fatty tissue.^29^ Fibrous tissue is among the least scattering of the tissue types whereas fatty tissues are highly scattering. Thus, both extremes of light scattering, which often dominate absorption effects in tissue, are represented. Simulations were conducted at 700, 800, and 900 nm, which are commonly used for PA imaging. In addition, simulations were conducted at 1064 nm which is commonly used due to the ubiquity of the Nd:YAG laser. To sample the space between tissue types, we also ran simulations at 800 nm in which the tissue composition was a linear combination of the fibrous and fatty tissue types in 20% increments. As suggested in the literature, all tissues were simulated with an anisotropy coefficient of 0.9^30^ and a refractive index of 1.4.^29^^–^^33^

**Table 1:** List of optical properties for all tissue type and wavelength combinations that were simulated.

Tissue type	Wavelength (nm)	Absorption coefficient (1/mm)	Scattering coefficient (1/mm)	Anisotropy coefficient	Index of refraction	References
Human and mouse geometry simulations						
Skin	700, 800, 900, 1064	0.042, 0.035, 0.031, 0.031	14.3, 15.9, 16.8, 16.8	0.9	1.4	29–33
Fatty tissue	700, 800, 900, 1064	0.099, 0.095, 0.096, 0.097	12.2, 11.3, 10.2, 9.09	0.9	1.4	29–33
Fibrous tissue	700, 800, 900, 1064	0.010, 0.016, 0.064, 0.076	2.31, 1.91, 1.63, 1.30	0.9	1.4	30,32–35
20% fatty, 80% fibrous tissue	800	0.032	3.80	0.9	1.4	29–35
40% fatty, 60% fibrous tissue	800	0.048	5.68	0.9	1.4	
60% fatty, 40% fibrous tissue	800	0.063	7.56	0.9	1.4	
80% fatty, 20% fibrous tissue	800	0.079	9.44	0.9	1.4	
Experimental validation						
Milk	1064	0.02	7	0.7	1.338	36–39
Gelatin	1064	0.012	0.05	0.85	1.5	40, 41

We also sought to investigate species-specific variations in the fluence distribution between mice and humans arising from differences in the thickness of their skin, which is highly scattering. The absorption and scattering properties of skin were taken from Ref. 29, as the optical properties between the species have been shown to be similar.^29^^,^^42^ The thickness of epidermis and dermis in humans is 1 to 4 mm,^29^ while in mice it is only 0.3 to 0.5 mm.^42^^,^^43^ Each tissue type and wavelength combination was simulated with a 2-mm skin layer to mimic human skin and a 0.3-mm skin layer to mimic the skin of mice. It was assumed that the fiber bundle was pressed flush with the skin, so no US gel or other coupling medium was included in the simulation volume.

For each species, tissue type, and wavelength combination, two sets of simulations were conducted. In the first set, the distance between the location of light emission and the center of the transducer, D, was held constant while the angle was varied. The distance, D, was set to 7 mm. This was chosen because it is close to the minimum possible given the size of the transducer used in our experiment and because many other transducers would be similarly sized. The angles were sampled from 20 deg to 70 deg in 12.5-deg increments, which spans the range of angles likely to be used. In the second set of simulations, the angle was held constant while the distance between the fiber bundle and the center of the transducer was varied. The angle was set to 45 deg since it is intermediate in the angles used in the prior simulation, and because we found from initial simulations that it commonly results in the highest fluence in the imaging plane. The distance, D, was varied from 5 to 13 mm. We used these distances because most transducers have a width greater than 10 mm, and because we found that the fluence drops off significantly beyond 13 mm. The reported fluence for each simulation was the fluence averaged over a 0.5  mm×20  mm rectangular region at the center of the imaging plane. A cubic volume with a length of 36 mm on each side and $10^{9}$ photons were used to simulate each condition. Finally, we compared the fluence delivered to the imaging plane when the mouse skin thickness was used and when the human skin thickness was used. This was conducted to determine the extent to which the difference in skin thickness contributes to lower fluence when translating from mice to humans, even before other factors such as greater imaging depth are considered. The maximum fluence at all fiber angles for the simulations in which the distance was held constant was calculated for each tissue type, wavelength, and species combination. The ratio of the maximum fluences between each mouse and human skin thickness simulation pair is calculated for each tissue type and wavelength combination.

### 2.3 Experimental Validation

#### 2.3.1 Experimental setup and analysis

The Verasonics system (Vantage 256™, Verasonics, Inc., Kirkland, Washington) was used for US and PA signal acquisition. The previously developed acquisition sequence, which incorporates a laser, the Verasonics system, and motorized stages,^44^^,^^45^ was used for acquisition of the US and PA data. The ultrasonic signals were acquired with a 128-element linear array transducer (Verasonics, Inc.; L11-4v) with a frequency bandwidth from 4 to 11 MHz. For US imaging, the center frequency was at 8 MHz and plane-wave compounding with 15 angles was utilized. PA excitation was achieved with a Nd:YAG pumped optical parametric oscillator laser (Opotek Phocus). The laser operated at 10 Hz with 7-ns pulses. The laser irradiation was delivered to the phantom by optically coupling the laser output to a 9-mm-diameter optical fiber bundle. The optical fiber bundle terminated in two rectangular bundles with dimensions of 25  mm×1.4  mm.

The phantom used for the experimental validation consisted of multiple 0.5-mm-diameter graphite rods embedded in a 6% gelatin (Sigma-Aldrich, G2500-1KG) phantom. Eleven rods were spaced 5 to 6 mm horizontally at depth increments of ∼2  mm [Fig. 1(d)]. The phantom was irradiated at 1064 nm with 40 mJ per pulse measured out of the single optical fiber bundle used in the experiment [Fig. 1(c)]. The transducer and optical fiber bundle were scanned across the graphite rods using a three-dimensional (3-D) positioning system (Newport; Model ESP301) in 0.2 mm steps, with 1 US and 1 PA image taken at each location, which was repeated four times for statistical analysis. The translation distance for 3-D scanning was 50 mm, so a total of 251 steps were required. One optical fiber bundle was sufficient to deliver laser irradiation to the rods [Fig. 1(c)] due to the symmetry of the dual-optical fiber bundle setup commonly found in the literature. This acquisition was repeated with the optical fiber bundle at each combination of three angles (20 deg, 40 deg, and 60 deg) and three horizontal distances (7.5, 11.5, and 15.5 mm) from the transducer, resulting in a total of nine acquisitions. Both the US transducer and the optical fiber bundle output were located 2 mm above the gelatin phantom and coupled to the phantom using milk, which approximates the scattering and absorption coefficients of skin at 1064 nm.

The 11 images taken directly above one of each of the 11 graphite rods were used to determine the PA intensity from the rod. The PA intensity from a single rod was calculated by averaging the PA signal across the rod in each image. The standard deviation for each of those PA intensities was calculated using the four acquisitions taken at each location. Since the absorption coefficient and Gruneisen coefficient would be the same for each of the graphite rods, the measured PA signal is directly proportional to the fluence at each rod. Thus, the PA intensity can be compared to the average fluence at the depth of each rod in the model.

However, in these experiments, the effect of US attenuation, obliquity, and the depth-dependent transducer focus would vary from rod to rod due to their placement at different depths in the phantom. To account for this, the US images of each rod were used to normalize the PA images in a manner similar to the work of Ranasinghesagara et al.^46^^,^^47^ Given that the rods are nearly identical, the same magnitude of US should be measured at each rod once the US attenuation, obliquity, and depth-dependent transducer focus are taken into account. Thus, the PA intensities were normalized using the US data. The US intensity for each rod was calculated by averaging the US signal over the rod in each of the 11 images. The US intensities were then normalized to the maximum US intensity. Since the attenuation, obliquity, and depth-dependent transducer focus would affect the ultrasonic signal twice during US acquisition (once for each direction of travel), the square root of the normalized US intensity for each rod was calculated. Finally, the PA intensity of each rod was normalized rod to rod by these values. These adjusted PA intensities, with their standard deviations, were plotted for each combination of angle and fiber distance.

#### 2.3.2 Experiment matched modeling parameters

To simulate the experiment, modeling was performed with parameters chosen to emulate the materials used in the experiment (Table 1). Thus, the volume for the Monte Carlo simulation consisted of a 2-mm layer with the optical properties of milk, whereas the remainder of the volume was assigned the optical properties of water to approximate the optical properties of the gelatin phantom. At 1064 nm, milk has an absorption coefficient near 0.02  1/mm and a scattering coefficient of 7.0  1/mm.^37^^,^^38^ It has a lower anisotropy coefficient of 0.7^36^^,^^37^ and an index of refraction of 1.34.^38^^,^^39^ The absorption^41^ and scattering coefficients^29^^,^^31^ of gelatin were approximated with the properties of water. At 1064 nm, gelatin has an anisotropy coefficient of 0.85 and a refractive index of 1.5.^40^ A cubic volume with a length of 36 mm on each side and $10^{9}$ photons were used to simulate each condition.

The model was evaluated to determine the fluence as a function of depth along the imaging plane of the transducer. The average fluence across voxels in a 0.5  mm×20  mm area was used for comparison to the experiment, since this is equivalent to the cross section of the rods. These values were plotted for each combination of fiber bundle angle and fiber distance.

### 2.4 Model Convergence

Across the imaging plane for each simulation, a 0.5  mm×0.5  mm kernel was used to evaluate the SNR (average fluence divided by the standard deviation of fluence) at each voxel. The SNR values were averaged across the imaging plane for each experimental condition to determine the average SNR for each simulation.

## 3 Results

### 3.1 Modeling Parameters

**Fig. 2:**
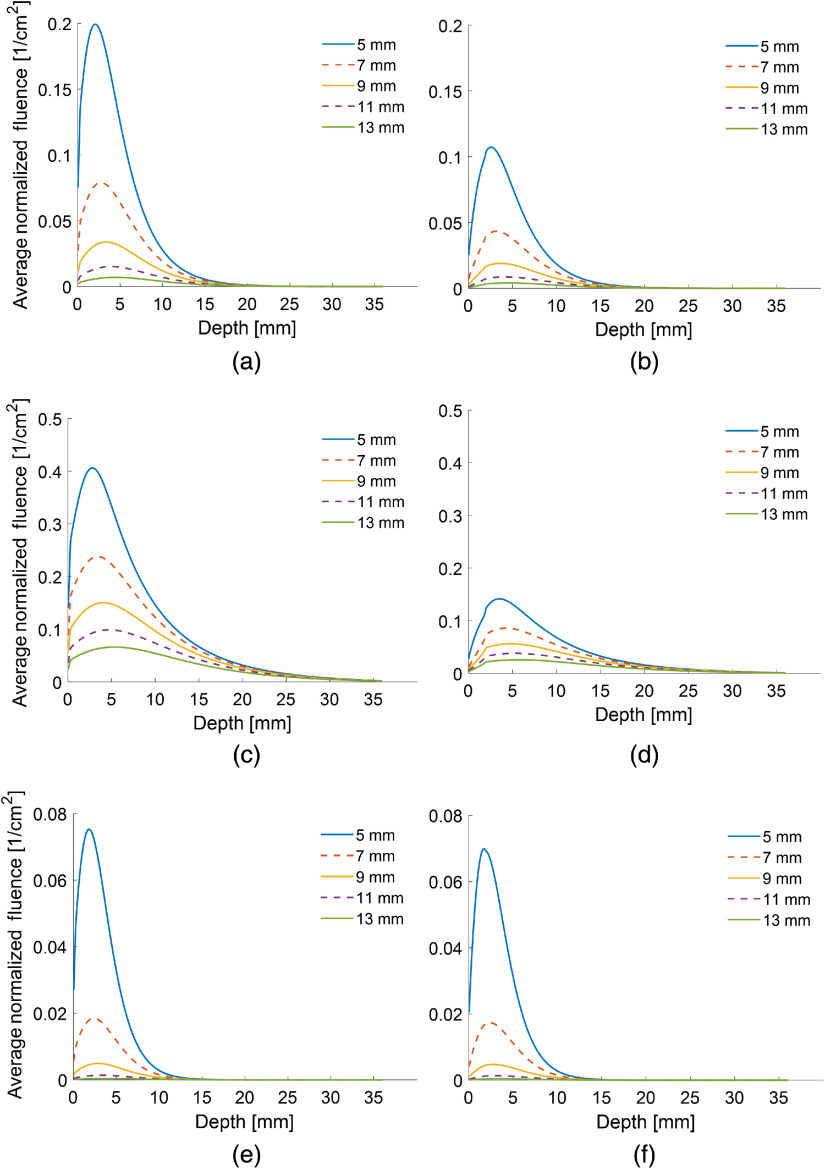
Plots of average normalized fluence versus depth. Simulations with a mouse skin thickness are on the left [(a), (c), and (e)], while simulations with the human skin thickness are on the right [(b), (d), and (f)]. Results for (a), (b) the 40% fat tissue at 800 nm; (c), (d) the purely fibrous tissue at 700 nm; and (e), (f) the purely fat tissue at 1064 nm are shown for the mouse and human skin thickness. Simulations were run with a constant angle of 45 deg and a variety of distances between the location of light emission from the optical fibers and the center of the transducer array. As expected, smaller distances between the location of light emission and the transducer result in higher fluences. Fluences are noticeably larger when the mouse skin thickness is used for the simulation, rather than the human skin thickness.

**Fig. 3:**
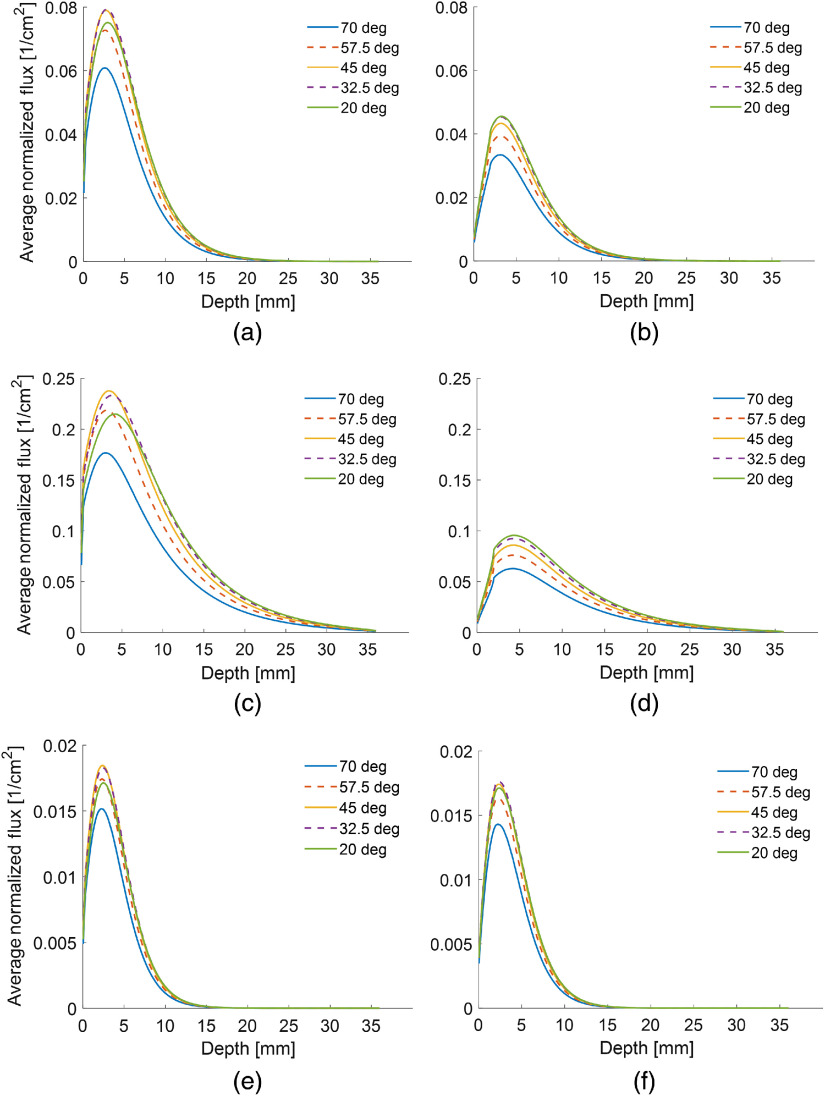
Plots of average normalized fluence versus depth. Simulations with a mouse skin thickness are on the left [(a), (c), and (e)], while simulations with the human skin thickness are on the right [(b), (d), and (f)]. Results for (a), (b) the 40% fat tissue at 800 nm; (c), (d) the purely fibrous tissue at 700 nm; and (e), (f) the purely fat tissue at 1064 nm are shown for the mouse and human skin thickness. Simulations were run with a constant distance of 7 mm between the location of light emission and the center of the US transducer, while varying the angle of light emission. In addition, shallower angles appear to deliver higher fluence across all depths for the human skin, while the optimal light emission angle is usually 45 deg when the simulations are run with the mouse skin thickness. Fluences are significantly higher when the mouse thickness is used instead of the human skin thickness. It is also highest for the least fatty tissues.

The fluence versus depth along the center of the imaging plane for several simulations with the mouse skin and human skin thickness is displayed in Figs. 2 and 3. Only the plots for the 40% fat tissue at 800 nm [Fig. 2–3(a) and 3(b)], the purely fibrous tissue at 700 nm [Figs. 2–3(c) and 3(d)], and the purely fat tissue at 1064 nm [Figs. 2–3(e) and 3(f)] are shown, although all results will be discussed. The results with the mouse skin thickness are shown on the left [Figs. 2–3(a), 3(c), and 3(e)] and results with the human skin thickness are shown on the right [Figs. 2–3(b), 3(d), and 3(f)]. The y axis is defined as the averaged normalized fluence. It is averaged because the fluence was calculated by averaging across the voxels in a 0.5  mm×20  mm area at the center of the imaging plane. It is normalized because the MCXLAB software automatically normalizes the fluence output by the amount of energy irradiated into the simulation volume. Fluence drops significantly as a function of depth in all simulations. For the fibrous tissue, it drops to zero near 35 mm at the end of the simulation volume, whereas for the highly scattering fatty tissue, it decreases to nearly zero at a depth of only 15 mm. As would be expected, the depth at which fluence reaches zero decreases monotonically as the fatty tissue component increases for the mixed tissue type simulations conducted at 800 nm. Fatty, and thus more highly scattering, bulk tissue results in lower fluences at the imaging plane. The peak of maximum fluence occurs between 2 and 6 mm for the majority of conditions that were simulated, no matter the angle of the fiber bundle.

For nearly all simulations, the fluence as a function of depth decreases by over an order of magnitude as the distance of the fiber bundle from the imaging plane increases (Fig. 2). The decrease was smaller in a few simulations (fibrous tissue at 700 and 800 nm) although the fluence still decreased by at least a factor of 5. The likely cause is that in these simulations, the scattering coefficient of the soft tissue is still low relative to other simulated tissue types, and the absorption coefficient in fibrous tissue is smaller than at 900 or 1064 nm.

Varying the light emission angle (Fig. 3) while holding the distance constant had a more complex effect on the peak fluence. For simulations with the fatty tissue, 45 deg was optimal for both humans and mice, with 32.5 deg giving a fluence within several percent of the maximum for all wavelengths. In fibrous tissue, 45 deg was optimal with the mouse skin thickness while 20 deg was optimal when the skin had the thickness of a human. For skin with the human thickness, 20 deg was also the optimal angle for most mixed tissue types at 800 nm. The exception was for 80% fatty tissue, in which the optimal angle transitioned to 32.5 deg. The variation between 20 deg and 32.5 deg was only a few percent at these conditions. For simulations with skin the thickness of a mouse, 45 deg and 32.5 deg were optimal and gave almost identical peak fluences. Across all the simulations in which the angle was changed, the peak fluence varied by 10% to 50% between the angle with the optimal fluence and the angle with the least light delivery. The variation was greatest when the scattering coefficient of the soft tissue was small.

Perhaps most importantly, there is significant variation in the fluence at the imaging plane between simulations with different skin thicknesses when all other conditions are held constant. Using the simulations in which angle was varied, the ratio of maximum fluence delivered when the mouse skin thickness was used versus the human skin thickness varied from 0.68 to 3.53 (Table 2). The ratio is greatest for the most fibrous tissue and increased with greater fibrous tissue content in the simulations with the mixed tissue composition. For the fatty tissue, the ratio dropped below 1 at wavelengths from 700 to 900 nm. Given the optical properties of tissue types commonly used for PA applications, ratios between 1.5 and 2 can be most commonly expected. This has clear implications for the translatability of PA studies conducted in mice, even before other factors, such as the greater imaging depth required in humans, are taken into consideration.

**Table 2:** Ratios of maximum fluence between the simulations with mouse and human skin thicknesses, for simulations in which the distance was held constant at 7 mm.

Fluence ratios of fatty and fibrous tissues by wavelength				
	700	800	900	1064
Fatty tissue	0.68	0.81	0.92	1.05
Fibrous tissue	2.49	2.74	3.18	3.53
Fluence ratios of mixed fatty and fibrous tissues by fat content				
	20% fat	40% fat	60% fat	80% fat
Mixed tissue, 800 nm	2.09	1.74	1.41	1.10

### 3.2 Experimental Validation

**Fig. 4:**
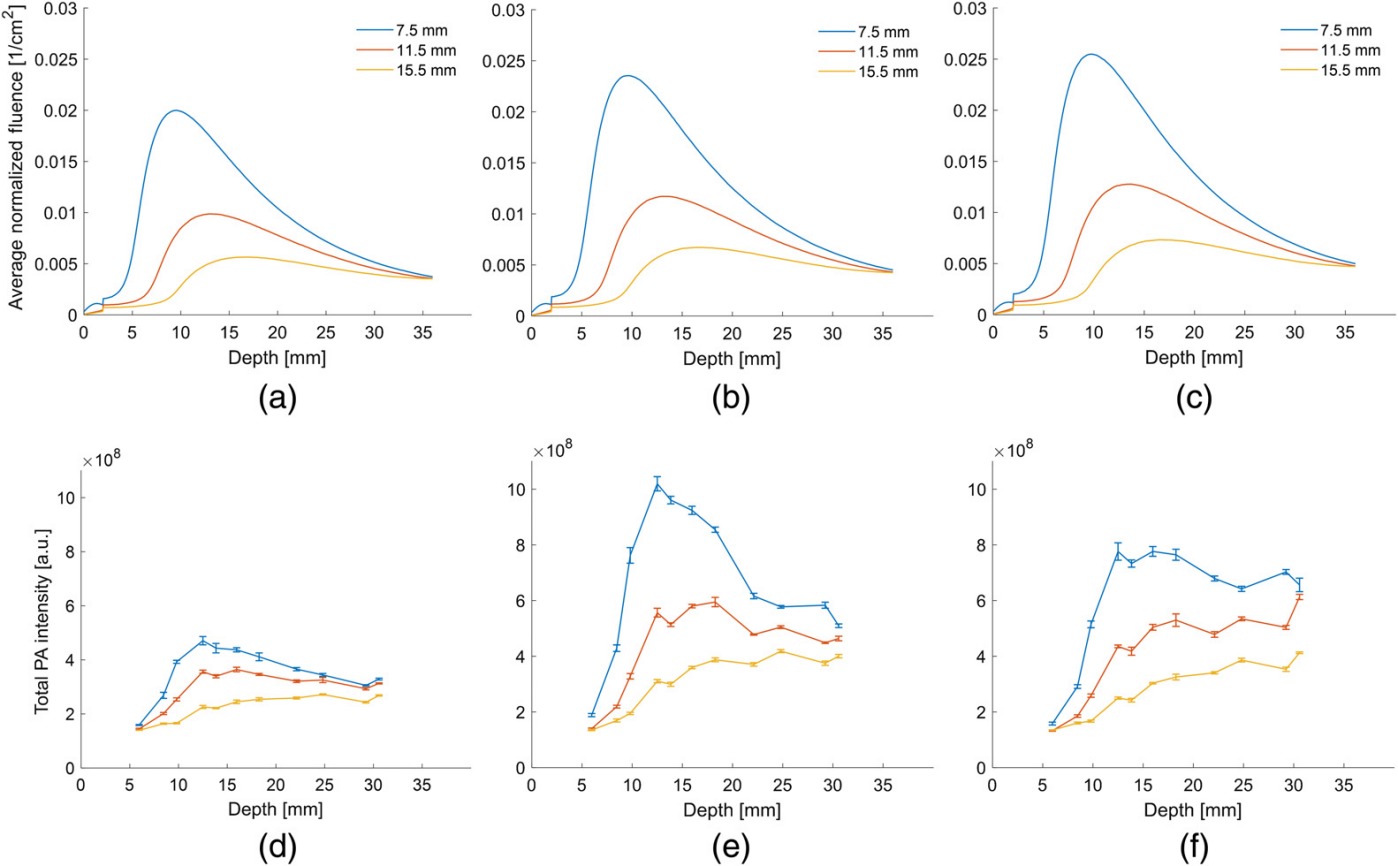
Comparison between (a)–(c) the experiment matched model and (d)–(f) the experimental results using the gelatin and graphite rod phantom. Simulations were conducted at 60 deg [(a) and (d)], 40 deg [(b) and (e)], and 20 deg [(c) and (f)]. In general, a smaller distance between the location of light emission and the transducer center results in higher fluences in both the experiment and simulations. Meanwhile, an angle of 20 deg or 40 deg is optimal for the model and experiment, respectively.

Results of the experimental validation and matching simulation are shown in Fig. 4. In both the experiment and the model, fluence varies significantly across all depths as the distance between the location of light emission and transducer center is increased. However, the change in the fluence is generally greater for the model than for the experiments. For the model, peak fluences between 20 deg and 60 deg varied by only about 25%, with the maximum peak fluence arising at the shallower angle of 20 deg. A similar difference for peak fluence with respect to light emission angle occurred when the location of light emission was farther from the transducer.

However, for the experiment, the maximum fluence occurred at 40 deg. In addition, variations in fluence were more significant. There was a 25% decrease in fluence when changing the light emission angle from 40 deg to 20 deg in the experiment with a 7.5-mm distance between the light emission and transducer center. At larger distances, the change generally ranged from 10% to 20%. A light emission angle of 60 deg resulted in fluences two times smaller than at a 40-deg angle when the distance was 7.5 mm, although this dropped closer to being two-thirds lower at 11.5 and 15.5 mm.

### 3.3 Model Convergence

The average SNR across the imaging plane was evaluated for each simulation. For the position and angle dependence studies in mice and humans, the average SNR across the imaging plane was 14.7. The minimum SNR for all conditions was 4.3. For the experimental phantom simulations, the average SNR was 28.8, with a minimum SNR of 15.0 for any single condition. The SNR values indicate that the photon count used for these simulations was sufficient. The smoothness of the processed data is evident in Figs. 2–3.

## 4 Discussion

In this paper, we have run a series of simulations with different system geometries, tissue types, wavelengths, and species characteristics to optimize fluence delivery for a PA imaging system. The difference in fluence distributions when either a mouse or human skin thickness is used indicate that there are differences in optimal design as well as the quantity of fluence that can be expected at a given depth depending on the skin thickness. First, in both models, the distance between the location of light emission from the optical fibers and the center of the US transducer has the most significant effect on the fluence delivered to the imaging plane. Minimizing this distance increases light delivery to the imaging plane. Variations of only several millimeters can result in changes in fluence of nearly twofold.

**Fig. 5:**
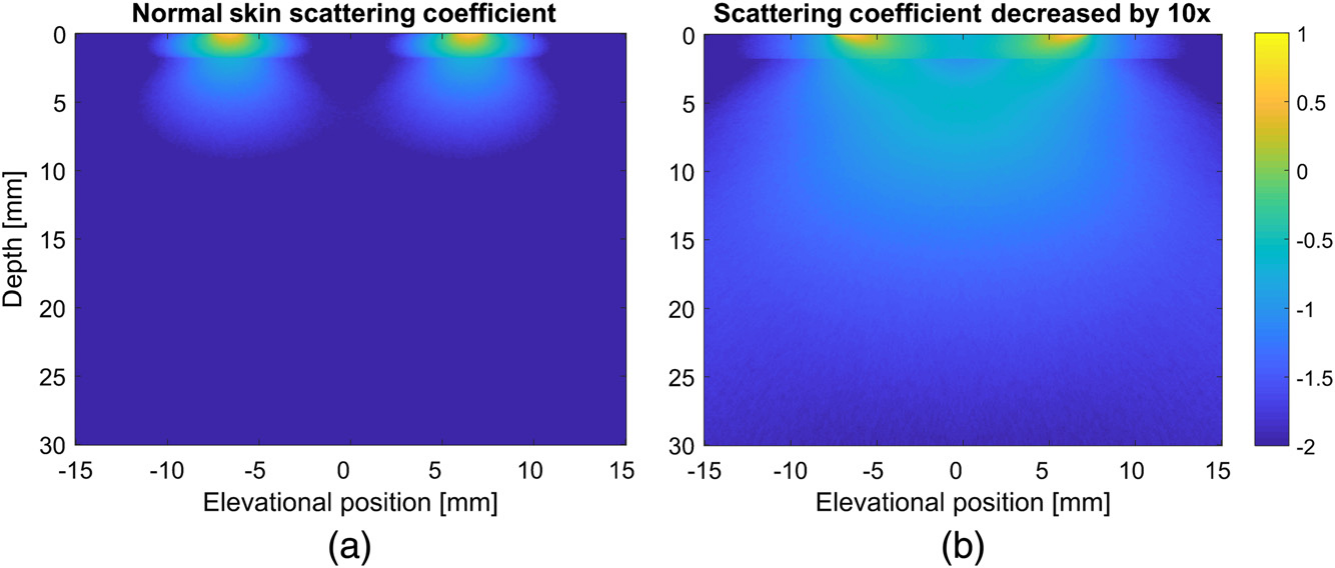
Color map of the fluence in a cross section of the simulation volume perpendicular to the center of the imaging plane. (a) A simulation with light emitted at a 60-deg angle through skin with a human skin thickness. (b) An identical simulation geometry with the human skin scattering coefficient decreased by a factor of 10. The difference in the fluence map clearly indicates the scattering effect of normal human skin thickness, which causes the photons to lose much of their ballistic directionality and instead have a more diffusive transport behavior.

Variations in the optimal angle were found to be dependent on the skin thickness and tissue composition. Angle had the greatest effect on fluence when the bulk tissue was more fibrous, and thus had a scattering coefficient several times smaller than the skin layer. For simulations with the mouse skin thickness, a 45-deg angle was optimal for most conditions with 32.5 deg often giving almost identical fluence. However, when the human skin thickness was used in the simulations, the optimal angle was instead 20 deg, with 32.5 deg sometimes giving similar results. The difference is due to the more complete scattering of light as it passes through the thicker 2-mm skin layer. For the human skin thickness, light is completely scattered such that the light propagation is diffusive rather than ballistic. This is illustrated in Fig. 5, which depicts a cross section perpendicular to the imaging plane. Light propagating through the 2-mm thick skin layer has lost its directionality despite having an angle of illumination of 60 deg [Fig. 5(a)]. However, when the skin scattering coefficient is decreased by a factor of 10, the angle of illumination is still evident, resulting in more light delivery to the imaging plane [Fig. 5(b)]. The mouse skin thickness is about 10 times smaller than the human skin thickness, so in the simulations the light maintains some of the directionality associated with the original illumination angle. As a result, the 45-deg angle, which directs the light closer to the imaging plane, is optimal.

Importantly, the extreme scattering in the thicker human skin layer results in lower fluences, even when using the optimal distance and angle configurations (Fig. 2 and 3). The modeling results indicate that the fluence will commonly be 50% to 100% higher in mice than in humans, and over 200% as high in fibrous tissue at 900 or 1064 nm. While this does not matter for imaging techniques developed for valuable preclinical applications, this should be strongly considered by researchers conducting techniques that are meant to be translated to humans, as most preclinical work is done in mouse models. While alternative methods for light delivery, such as using catheters or endoscopes, have been suggested that could circumvent this issue, they are not common.^48^ We have conducted our simulations using a variety of bulk tissue compositions and wavelengths, so that researchers can approximate the extent to which this will affect their specific applications. Researchers should expect that when translating their results to the clinic, fluences in human tissue may be several times smaller using the same equipment and pulse energies, simply because of the increased scattering in human skin.

The experimental validation was used to validate the trends found in the model. This was done by producing a gelatin phantom containing discrete absorbers at different depths and covered by a thin layer of milk, which approximates the optical properties of human skin. Then, the results of the experiment were compared to simulations of the experiments conducted on this phantom. The results indicate that the general trends found in the modeling above are consistent. In the model, there was a 2× to 4× difference in fluence when the distance between light emission from the optical fiber and the center of the US transducer was changed from 7.5 to 15.5. In the experiment, changing this distance also had the largest effect, with fluence decreasing by a factor of 2× to 3×. When changing the light emission angle, the model indicated that the fluence would vary by no more than 25% across the angles tested. In the experiment, the difference between 40 deg and 20 deg was 25%. For the 40 deg and 60 deg test, the change in angle had a larger effect on fluence at twofold. One potential source of error in these experiments is that some light, especially at extreme angles, could have been scattered upward and been absorbed after hitting the transducer. This possibility was not incorporated into our modeling, as the transducer was not present. In addition, the optical properties of the gelatin were approximated with those of water. Lastly, our simulations did not consider light reflecting off the boundaries of the simulation, which could have been possible at the gelatin–plastic and plastic–air interfaces.

Multiple researchers have investigated the optimal experimental geometry of similar imaging hardware in previous work. However, findings on the optimal angle for light delivery have varied. For example, Haisch et al.^15^ found that angles from 40 deg to 50 deg are optimal for depths from 10 to 25 mm, but that shallower angles are better for deeper targets. Sivasubramanian et al.^24^ found that a combination of the fiber–transducer distance, transducer–tissue distance, and light emission angle affect SNR in tissue. Sangha et al.^25^ found experimentally in a phantom that deeper focal depths resulted in higher depth penetration but at the cost of SNR in regions closer to the phantom surface, but this result did not carry over to *in vivo* experiments using a mouse. Conversely, Wang et al.^23^ found that smaller angles will result in monotonically higher fluence in the imaging plane at depths ranging from 5 to 30 mm. The divergent results can be explained by the presence or lack of a high scattering layer that replicates skin. For example, Haisch et al.^15^ did not include a skin layer in their phantom studies, while Sangha et al.^25^ used both a phantom without a skin-mimicking layer and validated the experimental work in a mouse. Sivasbramanian et al.^24^ also did not use a skin-mimicking layer. Thus, their finding that angle can be actuated to improve fluence in the imaging plane is consistent with the mouse model results in this work. In contrast, Wang et al.^23^ and other researchers^49^^,^^50^ included a 2-mm-thick skin layer in their simulations, and the results of those studies therefore correspond with the results in the human model used here. Thus, the differences in the effect of angle found between these researchers are explainable by the presence, or lack, of a scattering skin layer. Our modeling also indicated that reducing the fiber to transducer distance will increase the fluence delivered at the imaging plane. This finding was consistent with prior work in the literature.^15^^,^^23^^,^^51^

In this study, we evaluated the optimal design of an imaging system consisting of light emission and a commercial US transducer using the maximum fluence as the criterion for the optimal design. However, it has been found in the literature that in some instances, the increased background signal from other optical absorbers in the tissue can result in the optimal distance being farther away from the transducer.^49^^,^^52^ This effect is complex. The optimal distance can change, for example, when the transducer offset is increased. Also, studies on bright-field designs^53^^–^^56^ indicate that the increased clutter will not always offset the gain in fluence from moving the location of light emission closer to the imaging plane. In addition, the development of clutter removal techniques promises to reduce this effect.^57^^–^^62^ In general, the clutter will be a function of the chromophores in the tissue being imaged and the wavelength used for imaging, among other variables, which will vary from application to application. We recommend researchers carefully consider these factors or conduct direct testing when determining if a greater distance is appropriate for their application. We also did not consider studies in which the fluence is compensated to adjust for the decrease at greater depths, although techniques exploring this are in early development.^63^

Within the scope of this study, we examined only the distance between the location of light emission and the center of the US transducer and the light emission angle to optimize light delivery. However, other parameters are worth mentioning that have already been studied. One method used light reflectors on the transducer face or around the end of the optical fibers and transducer to reflect upwardly scattered light back into the tissue.^51^^,^^64^^–^^66^ However, experimental testing of the phenomena showed mixed results. When imaging arteries in mice *in vivo*, the reflector did not show the improvements found from modeling,^67^ although curved reflectors have given better experimental results.^64^^,^^66^ Periyasamy and Pramanik^51^ also considered different arrangements of the fiber bundles around the US transducer. We also did not consider the variation in fluence along the imaging plane, as this has been found previously to vary little with the length of the optical fiber bundle,^23^ nor did we consider the effect of offsetting the fiber bundle from the tissue surface as this has already been investigated.^51^ Finally, we did not consider the width of the optical fiber bundle in our modeling. This is because a larger width (with the same pulse energy) would effectively increase the average distance between the light source and the transducer bundle, which the results in this paper and the results of others^15^^,^^23^^,^^51^ have consistently shown result in a decrease in fluence. The other case, where the width of the optical fiber bundle is changed but the fluence is held constant rather than the pulse energy, results in an increase in depth penetration as has been shown previously.^68^

We also did not consider patient to patient or animal to animal variability in our work, since capturing all possible combinations is not possible. These could include differences in size and anatomy, as well as hair, fur (in mice), and skin pigmentation. Instead, we used bulk tissue at a variety of tissue compositions and wavelengths to achieve generalizable results. The goal is to give practical guidance to researchers, who can use the simulation results that best match the optical properties for their specific application as a starting point for designing their own imaging system.

In this work, we investigated optimal designs for the optical fiber and US transducer array combination that has become common for combined US and PA imaging. We focused on differences in optimal device designs using a variety of bulk tissue types, wavelengths, and system geometries. These results can be used by researchers as practical guidance for a variety of applications, because of the variety of tissue types and wavelengths that were simulated. Using the depth-dependent fluence delivered to the imaging plane as the criterion for evaluating the optimal design, we find that a smaller distance between the location of light emission from the optical fiber and the center of the transducer array results in the best light delivery for both species. However, the optimal light emission angle is dependent on the thickness of the skin layer and the tissue composition, with the former having the largest effect. We compare our results to the literature and find that the differences in skin layer thickness can explain variable findings for the optimal angles found in prior work. Perhaps most importantly, this work indicates that the same imaging system may deliver fluences several times smaller in humans than mice, which serves as a cautionary message for researchers currently developing new imaging techniques in mice models with the expectation that they will translate to humans.

## 5 Conclusions

In this study, experimental work and light transport modeling were completed to determine the optimal parameters for the design of a combined US/PA system consisting of an US linear array with external light delivery. Our simulations included a variety of tissue compositions, wavelengths, and equipment geometries. The effect of incorporating either a skin layer with the thickness expected in a mouse or with the thickness expected in a human was also investigated. With both skin thicknesses and all tissue type and wavelength combinations, reducing the distance between the light source and imaging plane has the largest effect on the light delivery to the imaging plane. However, the optimal light emission angle varied depending on skin thickness, tissue composition, and wavelength. In humans, shallower angles were found to increase fluence in the imaging plane across all depths. However, in mice an intermediate angle of 45 deg was most often optimal. The difference is shown to be mainly a result of increased scattering from the thicker human skin. The difference in the optimal fiber bundle angle between simulations with different skin thicknesses also explains variations in the results of studies by other researchers. Perhaps most importantly, our results indicate that an identical imaging system will deliver significantly less light to the imaging plane in humans than in mice because of the scattering effect of the thicker skin layer.
